# Health economics and integrated care: a growing and challenging relationship

**DOI:** 10.5334/ijic.2201

**Published:** 2015-06-17

**Authors:** Silvia MAA Evers, Aggie TG Paulus

**Affiliations:** Public Health Technology Assessment, Research School Caphri, Faculty of Health, Medicine and Life Sciences, Maastricht University, Maastricht, The Netherlands; Health Economics, Research School Caphri, Faculty of Health, Medicine and Life Sciences, Maastricht University, Maastricht, The Netherlands

In recent decades, the number of published health economic studies – especially economic evaluation studies – in the field of *integrated* care has increased substantially. One thesis summary has been published in the current issue of IJIC [1]. In general, these studies do not focus on developing innovative health economic methodologies, beyond the current state of art, but instead apply traditional methods usually developed within the medical field. The problem with this approach is that integrated care is often being evaluated with unsuitable health economic methods, as the main characteristics of integrated care models often differ from more traditional models of medical care. Consequently, a continued use of traditional health economic methods might result in invalid conclusions. This may hamper scientific research as well as policy-making on integrated care.

Several authors have discussed these issues, highlighting the fact that there are still many scientific challenges. As such the field of ‘health economics of integrated care’ is still in its infancy stage [1–6]. The key challenges in health economics arise from the distinctive features of integrated care, including continuous changes in the delivery of care, the lack of standardised or generalised outcomes or the presence of uncertain outcomes [7]. These and other characteristics make it difficult to develop adequate study designs, synthesise evidence, and measure, value and compare the costs and outcomes of different integrated care structures [8]. Health economists therefore encounter different problems and challenges when looking at integrated care.

To discuss these problems, challenges and the growing relationship between health economics and integrated care, a meeting was held during the congress of the International Foundation for Integrated Care (IFIC). The meeting took place in Brussels in 2014, with about 20 participants. Four challenging areas were identified and discussed: (1) searching literature and assessing the quality of the integrated care literature, (2) outcome measurement and valuation in integrated care, (3) determination of the costs of integrated care and (4) several other design and analysis aspects.

The first area of challenges is related to the search for and assessment of the quality of literature on integrated care. This subject is especially important for model-based economic evaluation studies. For modelling, these studies rely on a systematic synthesis of existing literature. This requires well-defined and clear strategies to search for the right literature and the presence of evidence-based checklists to determine the methodological quality thereof. With respect to integrated care, sensitive and specific search strategies as well as a quality assessment checklist are still lacking.

The second challenging area is the outcome measurement and valuation of integrated care. A proper measurement and valuation of integrated care requires the incorporation of its main characteristics. Among others, they include the fact that integrated care often has a multi-outcome focus instead of a focus on a single (clinical) outcome. Integrated care is often aimed at improving client satisfaction, access, affordability, sustainability and (thus) at improving the process of care. Moreover, integrated care arrangements do not only focus on the individual but also on the health and well-being of significant others. Next to this, the relevant domain of the outcome measurement for the client can vary when going through the integrated care chain. Clients, for instance, might value outcomes in the acute phase differently than in the after-care phase. Traditionally, in health economics, single (clinical) outcomes, quality of life or quality adjusted life years for single patients (often in a clinical phase or single stage) are used as an important measure to express outcomes. Given the specific characteristics of integrated care, a considerable methodological challenge in outcome measurement is thus to develop and/or establish sensitive generic outcome valuation methods aiming at providing accurate and relevant information on the outcomes of integrated care throughout the whole care chain, for both the client as well as his/her significant others.

The third range of challenges is related to the determination of the costs of integrated care. To properly calculate these costs, a cost method is required which is robust enough to incorporate particular features. These include, among others, continuous changes in the delivery of integrated care (so a heterogeneous production process), the absence of standardised outcomes (so no clear outputs) and the presence of many indirect costs (because of many coordinating activities and cooperative actions). Traditional cost methods are usually output-oriented and assume homogeneous production patterns, uniform standard outputs and the importance of direct costs only [7]. Given the features of integrated care, the challenge is therefore to develop and apply more process-oriented cost methods which also incorporate indirect costs (such as activity-based costing and transaction cost approaches) instead of the traditional more output oriented cost methods [3, 4, 7].

Finally, during the Brussels meeting, other design and analysis aspects were discussed. These relate to an adequate study design, such as a naturalistic study design instead of the randomised controlled trial, definition of comparators, dealing with missing data, sound statistical analysis and standardised guidelines for reporting scientific research on health economic approaches of integrated care.

In summary, during the last decades the methodological challenges related to health economics and integrated care have been highlighted by several experts [1–7]. Now the time has come to put the ideas on how to handle the identified challenges into action. This can be done by developing, adapting and improving innovative health economic methodologies for integrated care. To facilitate this development, currently the IFIC is establishing a Special Interest Group (SIG) in health economics and integrated care. The aim of this group is to discuss methodological challenges and to develop consensus and evidence-based health economic methodologies for integrated care. If you are interested to join this SIG, please contact Viktoria Stein (KST@euro.who.int).
